# A Navigation and Augmented Reality System for Visually Impaired People †

**DOI:** 10.3390/s21093061

**Published:** 2021-04-28

**Authors:** Alice Lo Valvo, Daniele Croce, Domenico Garlisi, Fabrizio Giuliano, Laura Giarré, Ilenia Tinnirello

**Affiliations:** 1 Dipartimento di Ingegneria, Università di Palermo, Viale delle Scienze, Ed. 9, 90128 Palermo, Italy; daniele.croce@unipa.it (D.C.); domenico.garlisi@cnit.it (D.G.); fabrizio.giuliano@unipa.it (F.G.); Ilenia.tinnirello@unipa.it (I.T.); 2 Consorzio Nazionale Interuniversitario delle Telecomunicazioni, Viale G.P. Usberti, 181/A, 43124 Parma, Italy; 3 Dipartimento di Ingegneria “Enzo Ferrari”, Università di Modena e Reggio Emilia, Via P. Vivarelli, 10, 41125 Modena, Italy

**Keywords:** navigation, visually impaired, computer vision, augmented reality, cultural context, convolutional neural network, machine learning, haptic

## Abstract

In recent years, we have assisted with an impressive advance in augmented reality systems and computer vision algorithms, based on image processing and artificial intelligence. Thanks to these technologies, mainstream smartphones are able to estimate their own motion in 3D space with high accuracy. In this paper, we exploit such technologies to support the autonomous mobility of people with visual disabilities, identifying pre-defined virtual paths and providing context information, reducing the distance between the digital and real worlds. In particular, we present ARIANNA+, an extension of ARIANNA, a system explicitly designed for visually impaired people for indoor and outdoor localization and navigation. While ARIANNA is based on the assumption that landmarks, such as QR codes, and physical paths (composed of colored tapes, painted lines, or tactile pavings) are deployed in the environment and recognized by the camera of a common smartphone, ARIANNA+ eliminates the need for any physical support thanks to the ARKit library, which we exploit to build a completely virtual path. Moreover, ARIANNA+ adds the possibility for the users to have enhanced interactions with the surrounding environment, through convolutional neural networks (CNNs) trained to recognize objects or buildings and enabling the possibility of accessing contents associated with them. By using a common smartphone as a mediation instrument with the environment, ARIANNA+ leverages augmented reality and machine learning for enhancing physical accessibility. The proposed system allows visually impaired people to easily navigate in indoor and outdoor scenarios simply by loading a previously recorded virtual path and providing automatic guidance along the route, through haptic, speech, and sound feedback.

## 1. Introduction

Nowadays, the determination of the position of a person or an object in space is fundamental for many applications and services. Many positioning systems already exist and the most well-known is the Global Positioning System (GPS). This type of system is very common and useful for many applications, such as in aviation, naval, and terrestrial fields. GPS technology is also integrated into most smartphones in order to provide various services, such as outdoor pedestrian navigation. Unfortunately, for some application scenarios like indoor environments, GPS does not work properly because it suffers in terms of precision or it might fail completely. This is due to signal attenuation that makes the received power too low for detection. For this reason, the research community has focused its attention on indoor positioning systems (IPSs), where GPS is not available.

There is a large variety of techniques to provide indoor positioning, which can be classified as (i) radio-frequency-based technologies [1,2], (ii) sensor-based technologies [3,4], and (iii) vision-based technologies [5,6]. For example, Wi-Fi-based positioning systems (WPSs), radio frequency identification (RFID), and ultrasound belong to the first category. In fact, they are methods that use the triangulation of RF signals or direct sensing. Conversely, dead reckoning belongs to the second category. More specifically, it is based on different inertial measurement unit (IMU) sensor readings, such as accelerometers, magnetometers, and gyroscopes. Finally, thanks to the pervasive availability of smartphones and digital cameras, vision-based solutions have been developed, opening the way to new augmented reality (AR) applications. However, many of these AR applications are being developed using some dedicated hardware [7,8], with little attention given to the requirements of people with special needs. Moreover, the cost of buying new dedicated hardware may become a barrier for the widespread diffusion of such technologies.

Instead, in this paper we exploit vision-based IPS technologies to design new navigation and AR services for visually impaired people, simply using common off-the-shelf smartphones. Indeed, smartphones are portable and affordable devices that are already in everyone’s pocket. In [6], we already presented ARIANNA, a system that uses the computer vision capabilities of common smartphones and is able to recognize a painted line on the floor, for guiding visually impaired users along predefined paths. Recently, ARIANNA has been enhanced with IMU sensors and is capable of working under varying environmental light conditions [9] as well as providing information on the surroundings, such as in [10]. However, the weak point of the ARIANNA system is the need to adapt the environment by installing a physical line on the floor, which might become a costly and time consuming task. Instead, in this work we design and implement a novel solution, called ARIANNA+, which avoids the need for the painted line. The system acts as a mediation instrument between user and environment, accompanying the blind person step-by-step and providing useful information on the environment upon request.

In more detail, ARIANNA+ exploits AR as a combination of real and virtual worlds, with real-time interaction, and accurate 3D registration of virtual and real objects to connect two points of interest (PoIs) without any physical painted line and safely guide the user along predefined paths. Then, once in the proximity of a PoI, ARIANNA+ provides access to content connected to that specific PoI; indeed, there is a clear distinction between the experiences of *physical access* to a chosen site, and *digital access* to the contents available, e.g., on a website, which represents a digital counterpart of the PoI. This distance between physical access and digital access can be reduced by means of the emerging augmented reality or mixed reality applications, which are becoming possible thanks to new generation smartphones and the ultra-responsive services of 5G networks. For this reason, in ARIANNA+ we add access to the digital contents associated with specific sites of interest through machine learning. The real-time association of digital contents to a physical space can combine the advantages of a real experience with those of personalized navigation, by means of digital mediation tools designed for responding to the particular needs of the user (e.g., translation of descriptions, enlargements, or different colors for the visually impaired, etc.) and for facilitating the access to the digital contents. This association obviously requires a user localization function (even in indoor spaces) for identifying the user environment and the point of view, as well as the identification of objects and persons for potential interactions in the surroundings.

ARIANNA+ is thus a novel navigation and AR system based on computer vision and machine learning technologies, which allows autonomous mobility of blind people in indoor and outdoor environments. In this paper, we describe the design and implementation of the two main components of ARIANNA+ based on AR and computer vision, which in our opinion can enable an innovative experience, and constitute the main contributions of this paper:

- a new localization system based on ARKit, responsible for guiding the user along pre-defined virtual paths;
- a machine learning identification mechanism, responsible for facilitating access to the digital contents associated with specific sites of interest.

Although these two components can be customized for many different use cases and can easily be generalized to other kinds of users, we specifically deal with the case of visually impaired users, for which both digital and physical access to the sites of interest require a special design. For this reason, we decided to implement a monument recognition use case.

The paper is organized as follows. After a brief introduction on the related work in Section 2, Section 3 gives a review of the ARIANNA localization system. Then, Section 4 presents the theoretical and technical descriptions of our new ARIANNA+ system. Section 5 presents the evaluation of the proposed system in terms of localization and tracking as well as monument identification performance. Finally, in Section 6 we draw some conclusions and discuss the future evolution of our work.

## 2. Related Work

Several approaches have been proposed to support visually impaired people moving safely and independently in an unknown environment. In this section we will briefly review some of the literature in the fields of both IPS technologies and of AI-assisted recognition of images, objects, and environments.

### 2.1. Indoor Positioning System

A plethora of indoor navigation systems has been developed based on different techniques or exploiting several of these simultaneously. While [11] provides a complete overview of navigation systems specifically designed for visually impaired people, we will provide here only a few examples of IPS technologies, separating them based on the main technique employed.

*Radio-frequency-based technologies.* Concerning radio technologies, WPS approaches are exploited in [1,12,13], while time of arrival (ToA) is presented in [2]. Moreover, a review of ultrasound methods is investigated in [14]. These systems, however, are not precise enough for guiding visually impaired people. For this reason, RF-PATH-ID [15] is based on disseminating passive RFID tags and using a dedicated reader to acquire information with the help of the user. Finally, the use of both radio and visual landmarks have been largely investigated, e.g., in [16] where specific landmarks are deployed on the PoI in the environment.

*Sensor-based technologies.* Conversely, IMU sensors such as accelerometers, magnetometers, and gyroscopes are accurate in the low–medium range, and can be exploited for pedestrian dead reckoning (PDR). However, for long distances, PDR suffers from measurement drifts due to noise [17]. In [3,18] an extended Kalman filter (EKF) is exploited to compensate for the drift. A method to reset the IMU bias can be found in [4], where knowledge of pedestrian movement is used. Despite their limitations, such dead reckoning solutions have been largely employed in real navigation systems [19] and in the Navatar [20] system. Other solutions adopt reference points provided by fingerprinting maps such as in [21,22].

*Vision-based technologies.* Cameras have been exploited in [23], as well as a vibrating belt giving navigation information, while the usage of smartphones is adopted in [24] to implement a vision-based navigation system for blind people. As discussed previously, common smartphones are also exploited in ARIANNA [6], where computer vision (CV) techniques are used to detect landmarks, such as colored tapes, painted lines, or tactile pavings deployed in the environment for guiding visually impaired users along pre-defined paths. A similar approach is adopted in [25] where two deep convolutional neural network (DCNN) models are presented in order to assist the mobility of blind and visually impaired people using a robotic guide dog. Unfortunately, as described below, this solution is not very efficient in terms of time complexity.

A further approach is presented in [26], where a novel system based on augmented reality and vibrotactile stimulation of the smartphone is proposed. The system uses a virtual target point that changes progressively. As feedback, smartphone vibration is used to notify the user when the target is in the view of the camera. However, the accuracy depends on the type of route and it is not very useful in the presence of obstacles.

Finally, optical self-tracking has been implemented in mobile AR headsets [5], but at the cost of adding extra hardware that might become expensive. Instead, in ARIANNA+ we exploit commercial off-the-shelf smartphones to build a virtual path that accompanies the blind person step-by-step, navigating the user towards the destination with constant and precise feedback.

### 2.2. Machine Learning for Image and Object Recognition

DCNNs are largely exploited in the context of image or object recognition and can be successfully exploited for identification of the surrounding environment. These neural networks are based on very interesting structures, called convolutional neural networks (CNNs), which are able to automatically extract the features characterizing different images or objects. In the specific context of digital services for tourism, for example, current research projects have mainly considered the problem of monument classification, such as the problem of identifying a special building architecture as a church, a palace, a bridge, or a tower. This is not a trivial task, especially because of the wide ranges of architectural solutions that have been proposed worldwide for implementing special classes of monuments [27]. Moreover, images of buildings in a real exploration can be partially captured by users, under varying environmental conditions (light, background, overlapping objects and people), which may complicate the identification process [28,29]. A project similar to ours, devised to identify a special site rather than a whole class of monuments, is presented in [30] by still working on image analysis.

Differently from previous approaches, as presented in [10], we consider the application of object detection algorithms to the problem of monument recognition. Object detection allows not only localization of the precise monument position within the image by means of a bounding box, but also recognition of small objects within a general background. This is very suitable for our scenario of visually impaired users, whose smartphones cannot be oriented easily towards the most relevant part of the monument. Since training an object recognition model from scratch takes a long time, we based our training mechanism on transfer learning, a popular method in the machine learning field, according to which a model developed for a specific problem is reused as the starting point for a new model.

## 3. The ARIANNA System

In this section we review the basics of the ARIANNA system. This overview will be useful in order to understand the operating principles and to understand the contributions of our new ARIANNA+ system.

### 3.1. Navigation Service

As mentioned, ARIANNA is a system developed with the aim of assisting visually impaired people in their autonomous navigation. ARIANNA is able to recognize a painted line on the floor under varying light conditions thanks to an integrated computer vision algorithm. Different computer vision functions are adopted in order to identify the painted line, although various constraints are present. Most notably: (i) the path identification has to be real-time for the users; (ii) the system has to have little impact on the lifetime of the smartphone battery. Therefore, the system must have low complexity for the identification of the line in real-time.

For the detection of the path, the geometry and the colors of the tape are exploited. In more detail, ARIANNA identifies the painted lines in the captured image, quantifies the slope of the lines and converts this slope into an absolute orientation of the user. Indeed, the map of the paths is given by a sequence of segments. To identify the slope of the line seen by the camera, three different steps are implemented: (i) filtering the image, for reducing the noise and the details of the image background; (ii) applying the Canny algorithm, for detecting the edges of the objects in the image; (iii) identifying the sub-set of edges that can be considered as lines using the Hough transform.

Along the path, the system also permits the finding of some points of interest by detecting landmarks (such as QRcodes or iBeacons) and retrieving location-based information. Finally, the system adopts tactile stimuli (vibration of the smartphone) to provide feedback. It has been shown that the current consumption of typical vibration motors has a limited impact on the battery life of commercial smartphones [31] and that the energy savings coming from switching off the screen are higher than the costs introduced by vibrational cues [32].

### 3.2. Tracking Service

In the ARIANNA system, the tracking service is used to compare the user position with the known map of the environment. This service incorporates information on the path (together with the compass and accelerometer data) and provides feedback with the phone vibration. In order to enhance the tracking performance, optical flow and filtering techniques are used, as described in [6]. Indeed, tracking systems generally have two problems:

- distance and direction estimation (DDE);
- estimation error reduction (EER).

In order to solve the DDE problem, well known CV algorithms for corner detection and optical flow, followed by linear affine transformation (widely available, e.g., from the OpenCV library) are adopted. Conversely, for the EER, the extended Kalman filter (EKF) and the weighted moving average (WMA) are used. In more depth, optical flow is used to estimate the movement of the camera (i.e., the smartphone of the user) by analyzing the frames flowing in front of it. Unfortunately, this method suffers from estimation errors that can accumulate, causing an increasing deviation from the real path, as described in [33]. In particular, together with the typical errors in the optical flow and affine transformation, the tracking estimation is affected by (i) changes of focus and/or exposure of the camera; (ii) low resolution, slow image capturing, etc.; (iii) image processing problems due to the camera (compression, anti-aliasing) or the person’s movement (blurring).

**Figure 1:**
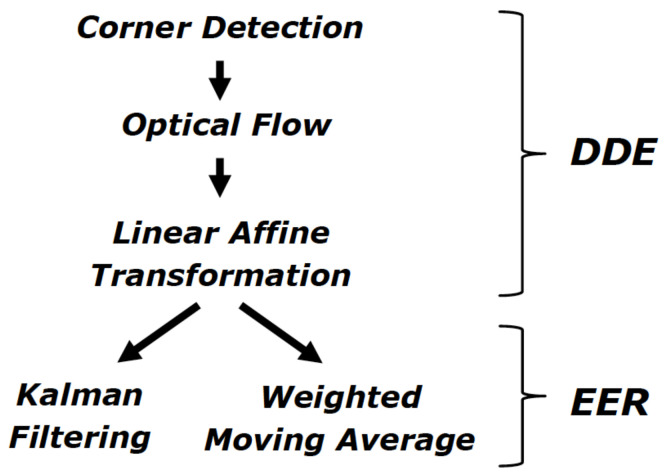
Tracking approach of ARIANNA system [6].

For this reason, it is very important to detect and filter such measurement errors. Therefore, two techniques are adopted: an EKF and a linear WMA filter. The EKF is used to estimate the state vector through observation measurements and for producing an estimate of the user position, speed, and heading. Conversely, the WMA filter reduces the error on the raw output of the CV functions. Figure 1 summarizes the techniques used for path estimation based on image processing.

## 4. The ARIANNA+ System

In this section we present the proposed new navigation system. Firstly, we describe the implementation of the virtual path, which is a substitution of the physical line, and provides the navigation and tracking services. Following this, we focus on object recognition. In particular, we decided to apply object recognition in a cultural heritage scenario, in order to assist visually impaired people in having a more accessible cultural experience.

### 4.1. Methodology: The Virtual Path

#### 4.1.1. Navigation Service

In many scenarios it is difficult to implement a physical path dedicated to visually impaired people, and even installing a simple line painted on the floor of cultural heritage sites can be a problem. Indeed, any kind of path or line applied on a precious historical pavement or in some important cultural context might not be appropriate. To overcome these difficulties, in ARIANNA+ we exploit the potential offered by augmented reality algorithms. Instead of a physical path, we apply a virtual path on the floor that is only visible through the smartphone. Thus, the methodology used in ARIANNA+ is similar to the ARIANNA system and the detection algorithm for the line is the same, as already reported in previous papers [6,9]. The difference in ARIANNA+ is the virtualization of the line on the floor (using augmented reality concepts), which reduces the economic cost of the system and simplifies the deployment in some urban or cultural heritage contexts, where it is not possible (or not allowed) to paint a real line on the floor.

In particular, in ARIANNA+ we implement a virtual line using Apple iOS tools and XCode, the integrated development environment (IDE) for developing software for iPhones, as well as two libraries for AR implementation, namely ARKit and SceneKit. ARKit is a framework that provides and processes sensor data necessary for AR experiences, while SceneKit is a 3D graphics framework that is useful for creating 3D scenes. These two libraries are used to analyze the environment and build the virtual path, respectively.

**Figure 2:**
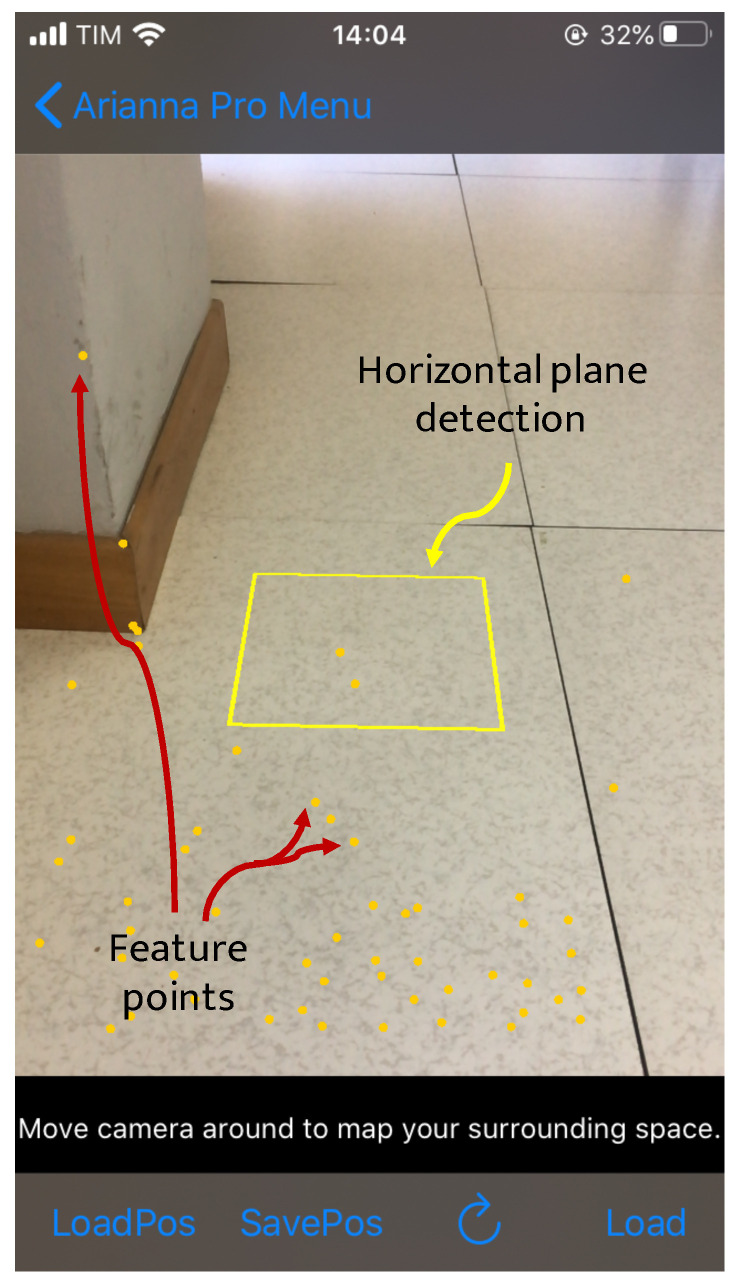
Screenshot of the feature points and horizontal plane detection through *hitTest*.

The first key component to understand the 3D world around the user is the *hitTest* method, which is able to search for real-world objects in the captured camera image. This method is used for finding the floor, i.e., a horizontal plane, and the feature points, as shown in Figure 2.

**Figure 3:**
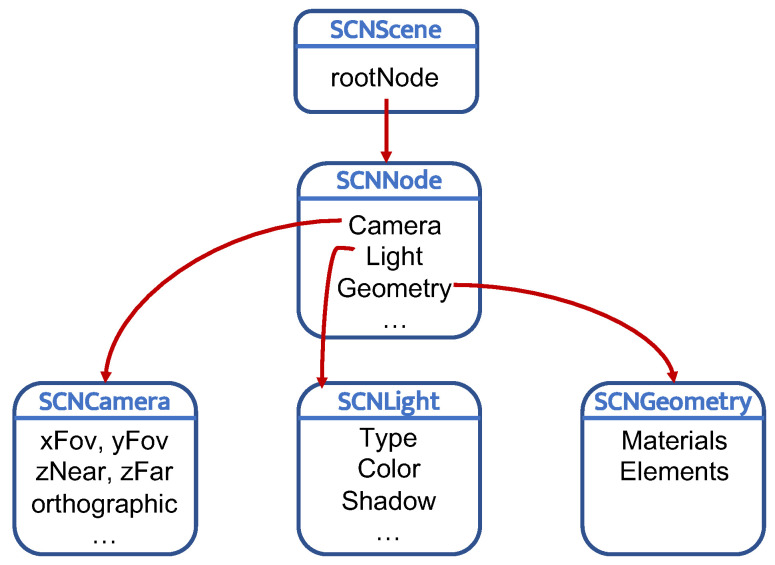
High level architecture.

The other key components for creating the virtual path are SCNScene, SCNNode, and SCNGeometry, all included in the SceneKit library. SCNScene is a container for the node hierarchy and attributes that together represent all the visual elements; SCNNode is a structural object of a scene that represents a position in a 3D coordinate space to which it is possible to attach geometry, lights, and cameras; SCNGeometry is a 3D shape that can be displayed in a scene and can be attached to an SCNNode. Figure 3 shows a high-level design of the interaction between these components. From a conceptual point of view, an SCNNode with its SCNGeometry are initialized and updated at every step.

Finally, the whole path within the 3D world of the scene is saved as map.

Thanks to these tools, we can create a virtual line that is able to guide visually impaired people for autonomous and flowing navigation. Along the path, the user receives a tactile stimulus when walking along the virtual line and is accompanied step-by-step guidance in the right direction with precise feedback. Indeed, visually impaired people cannot see the line on the smartphone, but they can feel the tactile vibration when the line is located at the center of the camera.

#### 4.1.2. Tracking Service

**Figure 4:**
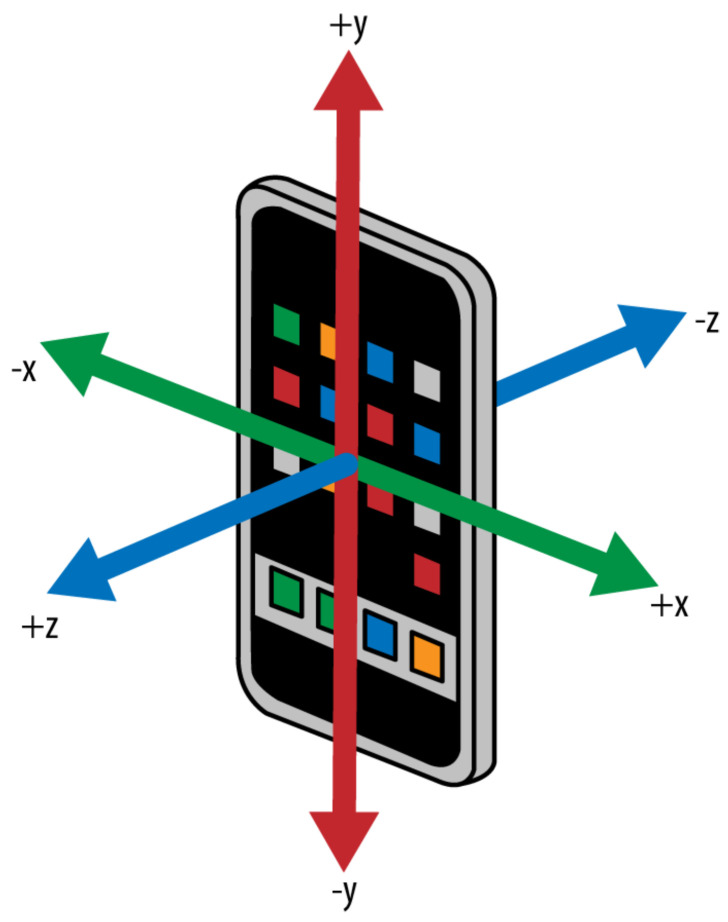
Camera coordinate system.

Regarding the tracking service, it is important to highlight the basic requirement for any AR experience: the ability to create and track correspondence between a real-world space and a virtual space where it is possible to add some virtual content. When virtual content is added together with the image captured live by a camera, the user experiences augmented reality, i.e., the illusion that the virtual content is part of the real world. In all AR experiences, ARKit uses world and camera coordinate systems following a right-handed convention: the y-axis points upward, the z-axis points toward the viewer and the x-axis points right, as depicted in Figure 4. Session configurations can change the origin and orientation of the coordinate system with respect to the real world. Each anchor in an AR session defines its own local coordinate system, also following the right-handed, z-towards-viewer convention. To create a correspondence between real and virtual spaces, ARKit uses a technique called visual-inertial odometry. This process combines information from the iOS device’s motion-sensing hardware with computer vision analysis of the scene visible to the device’s camera. ARKit recognizes notable features in the scene image, tracks differences in the positions of those features across video frames, and compares that information with motion sensing data. The result is a high-precision model of the device’s position and motion. The ARKit world tracking also provides the possibility to analyze and understand the contents of a scene. For example, if the planeDetection setting is enabled in the session configuration, ARKit detects flat surfaces in the camera image and reports their positions and sizes. It is possible to use ray-cast results or detected planes to place or interact with virtual content in the scene. Moreover, world tracking correlates image analysis with device motion. ARKit develops a better understanding of the scene if the device is moving, even if the device moves only subtly. Excessive motion—too far, too fast, or shaking too vigorously—results in a blurred image or too much distance for tracking features between video frames, reducing tracking quality. Finally, the ARCamera class provides tracking state information, which can be used to tell a user how to resolve low-quality tracking situations.

### 4.2. Environment Recognition: The Case of Cultural Heritage

We provide information on the surrounding environment by solving the object detection problem. In particular, as presented in [10], we focus on the specific use case of recognition of touristic and cultural heritage sites, such as monuments and churches. Indeed, these buildings are important for commemorating people and/or events, and are also common destinations for visually impaired people. In order to make the experience for blind people more accessible, we decided to search for a solution for automatic monument recognition. The idea is to support the access with the digital contents (voice description, sounds/music, etc.) associated to the physical building.

The idea is that the user, following the virtual path in both indoor and outdoor spaces, can change the orientation of the smartphone camera from the floor to the front space. This operation could be relatively easy for low-vision people (who can identify a building, without perceiving the details), but could also be possible for blind people, assisted by means of vocal messages suggesting the correct orientation once in the proximity of the PoI. Indeed, using ARIANNA+ (or ARIANNA) blind people know exactly where they are and when they have to point the smartphone at the surrounding monuments.

In any case, for the purpose of the environment recognition, the acquired images are in general of different qualities, with the monument located at any possible position within the image and, in some cases, only partially captured by the image. We thus analyzed various methods for object detection including region-based convolutional neural networks (R-CNN), faster-RCNN [34], single shot detector (SSD) [35] and you only look once (YOLO). R-CNN and faster R-CNN are composed of two stage detectors: the first stage identifies a subset of regions of interest in an image that might contain an object, while the second stage is used for object classification and bounding-box regression. The first stage in R-CNN is a slow object detection algorithm called selective search; faster R-CNN instead uses a very small convolutional network called a region proposal network to generate regions of interest. Conversely, SSD and YOLO are methods that consider detection as a regression problem and they are composed of only one stage detector. YOLO has the best performance in terms of training time, but not the best in terms of accuracy.

Thus, we compared the performance of different structures, based on the combination of multi-stages; namely, faster R-CNN and SSD in combination with Inception v2 [36] and ResNet [37]. We compared three different object detection structures (or models):

- Model 1: faster R-CNN, Inception v2;
- Model 2: faster R-CNN, Inception v2, ResNet;
- Model 3: SSD, Inception v2.

**Figure 5:**
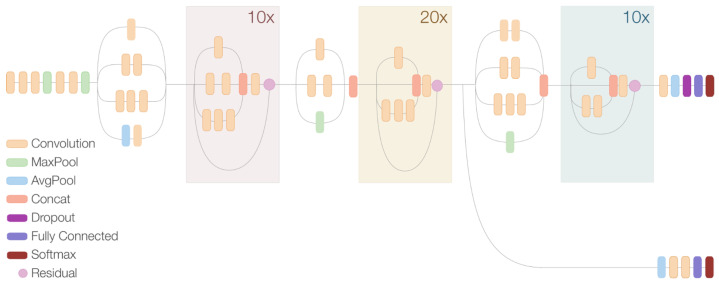
Faster R-CNN Inception ResNet v2 neural network layers.

As mentioned above, all the CNNs considered in our design are composed of different feature extractors: faster R-CNN, Inception v2, ResNet, and SSD. The main layers in a CNN are the convolution layer, the pooling layer, and the fully-connected layer. The primary purpose of a convolution layer is to extract features from the input image through a set of independent filters. The function of a pooling layer is to progressively reduce the spatial size of the representation, in order to reduce the amount of parameters and computation in the network. Finally a fully connected layer takes the end result of the convolution/pooling process and reaches a classification. An example of the composition of a CNN is shown in Figure 5.

## 5. Experimental Results

### 5.1. Navigation and Tracking

**Figure 6:**
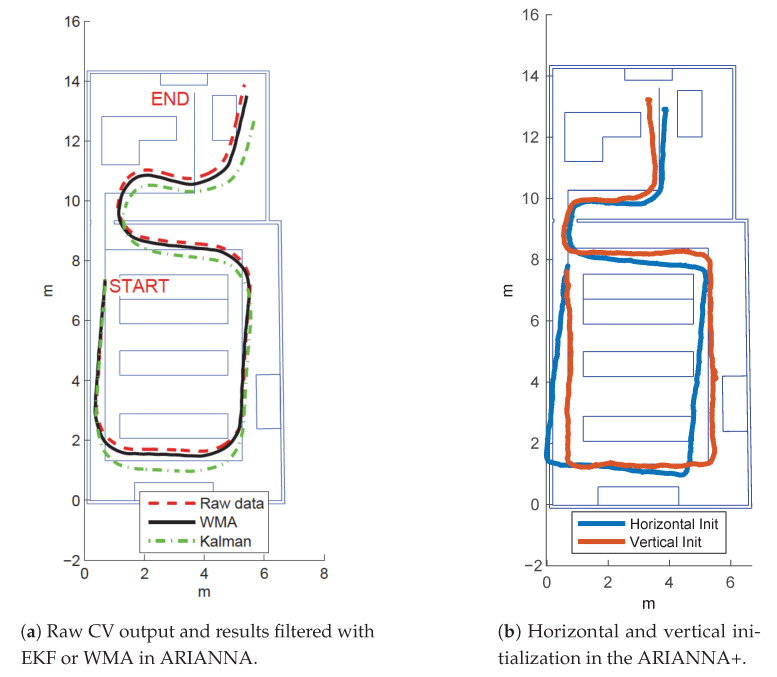
Tracking performance comparison using the previous ARIANNA system (a) or the proposed ARIANNA+ system (b) using different configuration options.

**Figure 7:**
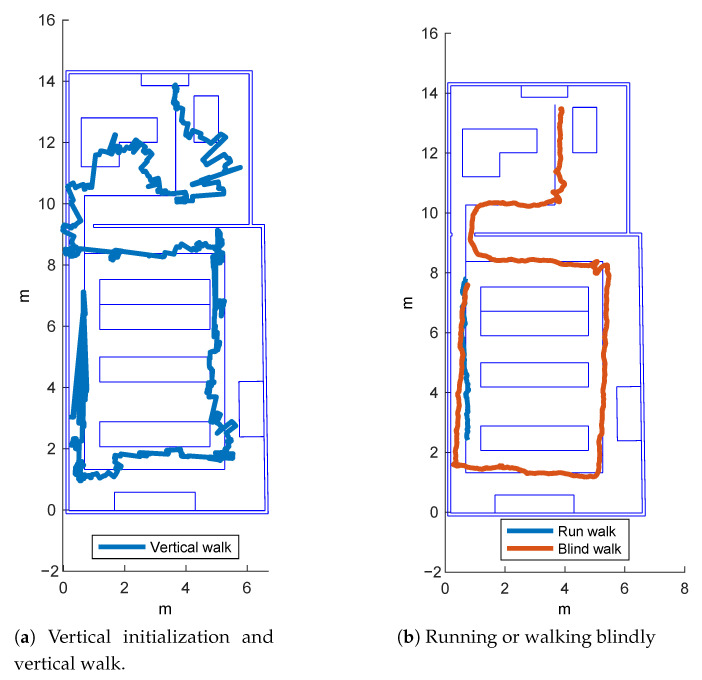
Tracking performance in ARIANNA+ with vertical initialization and walk, running or walking blindly.

In this section, we compare the ARIANNA system performance presented in [6] with the new navigation and tracking service implemented in ARIANNA+. In order to understand the accuracy of our new AR tracking method, we show the results of the different experiments carried out in a demo setting in a laboratory located in our campus. The path goes around the working desks and, through a door, enters a second room for a total length of 32.4 m. The rationale behind the testing environment was to create a path with a sufficiently high number of turns, but with a particular distance between them, to show the possible divergences of the algorithms. Moreover, the path needed to be sufficiently long so that a real user could walk on it giving rise to a real experiment. We created a 2D map of the environment expressed in Cartesian coordinates (X,Y), given in millimeters. The map shows in blue solid lines the obstacles (walls, tables, desks, etc.) and the physical path built in the laboratory. We considered three different paths and, for each of these paths, we tested the techniques previously described based on optical flow, and enhanced with an extended Kalman Filter (EKF) and a weighted moving average (WMA) for ARIANNA, as well as the new ARIANNA+ algorithms based on ARKit. For the sake of simplicity, we show here the results obtained following the most complex path. Figure 6a shows the results obtained with ARIANNA using the raw optical flow and the EKF and WMA methods. From the figure, it is clear that even with the application of complex filtering, the tracking estimation performance of ARIANNA is usually quite limited. Conversely, Figure 6b shows the tracking performance of the ARIANNA+ system with two different initialization methods. In particular, the difference between the two results stands in the initial position of the smartphone with respect to the floor: vertical initialization means that the smartphone accesses the camera when the initial position is perpendicular to the floor. Instead, horizontal initialization means that the position of the smartphone is parallel to the floor (flat), with a limited view of the surrounding environment. Indeed, when the smartphone is perpendicular to the floor, the camera can capture the environment better and compute the position more precisely relative to the surrounding objects, while these kinds of measurement are not as accurate when the initialization is parallel to the floor. For this reason, in the following experiments we always initialized the system with the vertical position, even if the phone was successively used flat to follow the virtual path. Indeed, another experiment consisted of initializing the camera vertically with respect to the floor, and then leaving the phone in the vertical position during the walk. Figure 7a shows the estimated tracking position obtained, which clearly has low accuracy. Therefore, we conclude that the best performance is obtained by first using vertical initialization and then holding the smartphone horizontally when following the virtual path.

Finally, Figure 7b depicts the tracking performance of the system in two different scenarios: when the user runs and when the virtual line is followed by a visually impaired person. The Figure shows that the blind person is able to successfully and safely follow the path even on the sharp turns, while when the user is running, even with the vertical initialization, the tracking is lost approximately at the first turn.

### 5.2. Monument Recognition

For the monument recognition, we evaluated the detection performance of different models for the recognition of monuments which are part of the UNESCO Arab–Norman itinerary in Palermo, Italy, a wonderful Mediterranean city in the island of Sicily. We adopted a transfer learning approach for training. More specifically, our neural networks were pre-trained by means of the Common Objects in Context (COCO) data set, a large-scale data set containing 1.5 million object instances and more than 200,000 labeled images. Models have been trained to recognize 10 monuments (i.e., theaters and churches) of different sizes and/or structures. The input dataset consists of 1572 photos (more than 100 pictures per monument), which have been explicitly taken for this purpose. We chose different poses, weather, and light conditions and with the presence of objects or people in the background. Finally, all the pictures were down-sampled in order to satisfy the training system requirements. For each image, we selected the exact location of the monument within the image, through LabelImg, a graphical image annotation tool, to label images for bounding-box object detection, and we associated the exact label. TensorFlow Object Detection API, an open source framework developed by Google, was used for training our three pre-trained neural networks. In particular, we used 80% of the images for training and the remaining 20% for evaluation. The networks were fine-tuned running 7500 consecutive iterations. During the training, we generated a log file for listing the labels and three new different models with the final weights. We trained all the three proposed structures with a common laptop (i.e., i7 processor, 16 GB RAM and without using any powerful GPUs) and the training times were about 11 h for the second structure and 3 h for both the first and the third structures.

**Figure 8:**
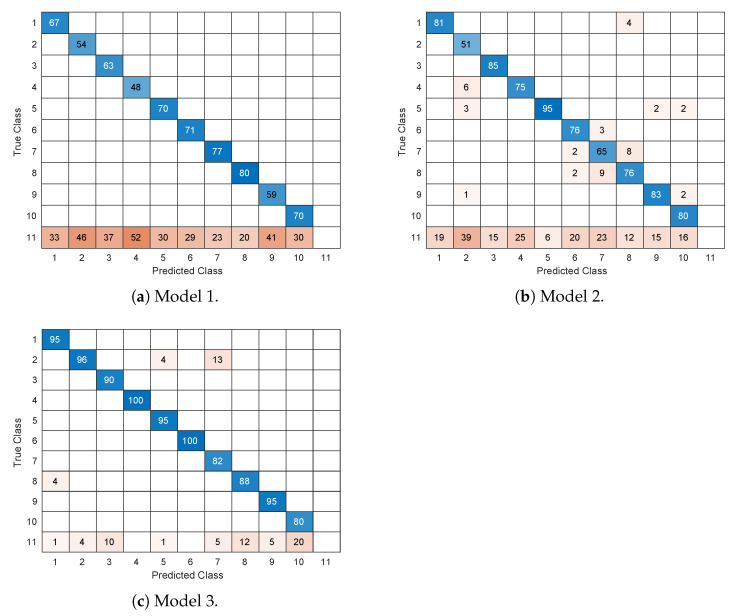
Confusion matrix of the three models considered.

Once the neural networks had been trained, we evaluated the results taking into account confusion matrices for each model. Precisely, the horizontal rows represent the ground truth, i.e., the correct monument label of the image, while the vertical columns represent the percentage of predictions corresponding to each possible monument in the set. In the last row, we also specify the percentage of times in which the monument was identified as different from any other monuments in the set. Figure 8a–c show three confusion matrices corresponding to the three different structures or models considered in our work. From the results, it is evident that the last structure, namely the one corresponding to the usage of SSD and Inception v2 (model 3), despite its simplicity and prompt training times, provided the best results. In most cases, the monument was correctly identified in more than 90% of the cases.

**Figure 9:**
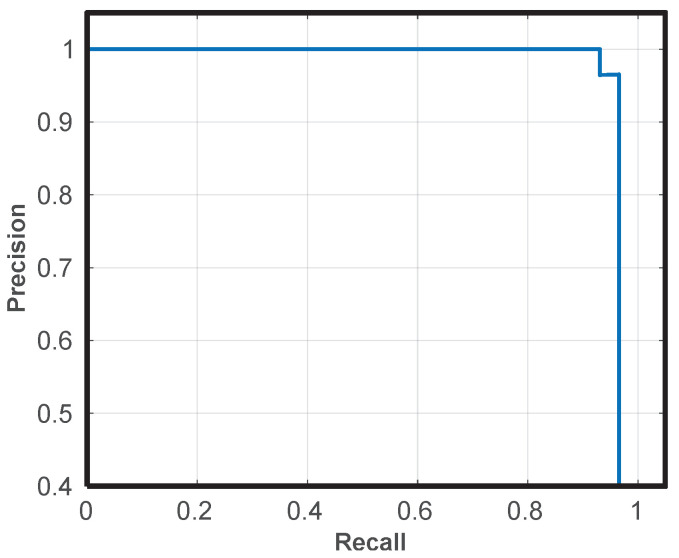
Precision vs Recall curve of St. Maria Valverde Church.

There are two other important metrics that can be evaluated: precision and recall. Precision refers to the percentage of results that are relevant; recall refers to the percentage of total relevant results correctly classified by an algorithm. For example, Figure 9 shows the precision vs. recall curve for St. Maria Valverde Church. This further demonstrates the good performance of model 3.

(1) $$\begin{array}{c} Precision=\frac{True\;Positives}{True\;Positives+False\;Positives};\\ Recall=\frac{True\;Positives}{True\;Positives+False\;Negatives} \end{array}$$

We can conclude that SSD Inception v2 for our points of interest in the Arab–Norman itinerary is the best solution for the identification of the monument.

## 6. Conclusions

In this paper we presented ARIANNA+, a smartphone-based solution to help visually impaired people navigate along predefined paths. Differently from the previous ARIANNA system, the newly proposed ARIANNA+ eliminates the need for any physical support thanks to AR technology, which we have exploited to build a completely virtual path. Moreover, the new solution offers to the users the possibility of enhanced interactions with the surrounding environment, through convolutional neural networks (CNNs) trained to recognize objects or buildings, thus enabling access to digital contents associated with them. By using a common smartphone as a mediation instrument with the environment, ARIANNA+ leverages AR and machine learning for enhancing physical accessibility. The proposed system allows visually impaired people to easily navigate in indoor and outdoor scenarios simply by loading a previously recorded virtual path and providing automatic guidance along the route, through haptic, speech, and sound feedback.

As future work, we are working on the extension of the proposed system. In particular, we are considering the integration of wearable devices, e.g., exploiting camera (vision) sensors and smart-watch (vibration) devices, in order to generate a more comfortable experience for visually impaired people. Moreover, we plan to perform experiments involving blind people, in order to better assess the usability of the proposed system. Since, from the user point of view, ARIANNA+ works in exactly the same way as ARIANNA, we conjecture that the results should be about the same as the ones presented in [38].
